# Fragment-Based Screening Maps Inhibitor Interactions in the ATP-Binding Site of Checkpoint Kinase 2

**DOI:** 10.1371/journal.pone.0065689

**Published:** 2013-06-12

**Authors:** M. Cris Silva-Santisteban, Isaac M. Westwood, Kathy Boxall, Nathan Brown, Sam Peacock, Craig McAndrew, Elaine Barrie, Meirion Richards, Amin Mirza, Antony W. Oliver, Rosemary Burke, Swen Hoelder, Keith Jones, G. Wynne Aherne, Julian Blagg, Ian Collins, Michelle D. Garrett, Rob L. M. van Montfort

**Affiliations:** 1 Cancer Research UK Cancer Therapeutics Unit, Division of Cancer Therapeutics, The Institute of Cancer Research, Haddow Laboratories, Sutton, Surrey, United Kingdom; 2 Division of Structural Biology, The Institute of Cancer Research, Chester Beatty Laboratories, Chelsea, London, United Kingdom; University of Pittsburgh School of Medicine, United States of America

## Abstract

Checkpoint kinase 2 (CHK2) is an important serine/threonine kinase in the cellular response to DNA damage. A fragment-based screening campaign using a combination of a high-concentration AlphaScreen™ kinase assay and a biophysical thermal shift assay, followed by X-ray crystallography, identified a number of chemically different ligand-efficient CHK2 hinge-binding scaffolds that have not been exploited in known CHK2 inhibitors. In addition, it showed that the use of these orthogonal techniques allowed efficient discrimination between genuine hit matter and false positives from each individual assay technology. Furthermore, the CHK2 crystal structures with a quinoxaline-based fragment and its follow-up compound highlight a hydrophobic area above the hinge region not previously explored in rational CHK2 inhibitor design, but which might be exploited to enhance both potency and selectivity of CHK2 inhibitors.

## Introduction

Checkpoint kinase 2 (CHK2) is a serine/threonine kinase crucial in the activation of signal transduction pathways involved in the cellular response to DNA damage caused by external agents [1], [2], [3], [4]. In response to double strand DNA breaks, CHK2 is activated through initial phosphorylation on Thr68 by the DNA damage sensor ataxia-telangiectasia mutated (ATM) [5], [6] and subsequent trans-autophosphorylation on Thr383 and Thr387 and cis-autophosphorylation on Ser516 [7], [8], [9], [10]. In its fully activated state CHK2 is known to phosphorylate a variety of substrates involved in DNA-repair, cell cycle control and apoptosis. For example, CHK2 phosphorylation of BRCA1 promotes the repair of double strand DNA breaks [11], while phosphorylation of the transcription factor forkhead box protein M1 enhances homologous recombination and base excision repair mechanisms [12]. Alternatively, CHK2 promotes apoptosis by phosphorylation of the transcription factor E2F1 [13] and by phosphorylation of the p53 interaction partner HDMX, which stabilises p53 and results in a G1 cell cycle arrest and cell death [14], [15].

**Figure 1:**
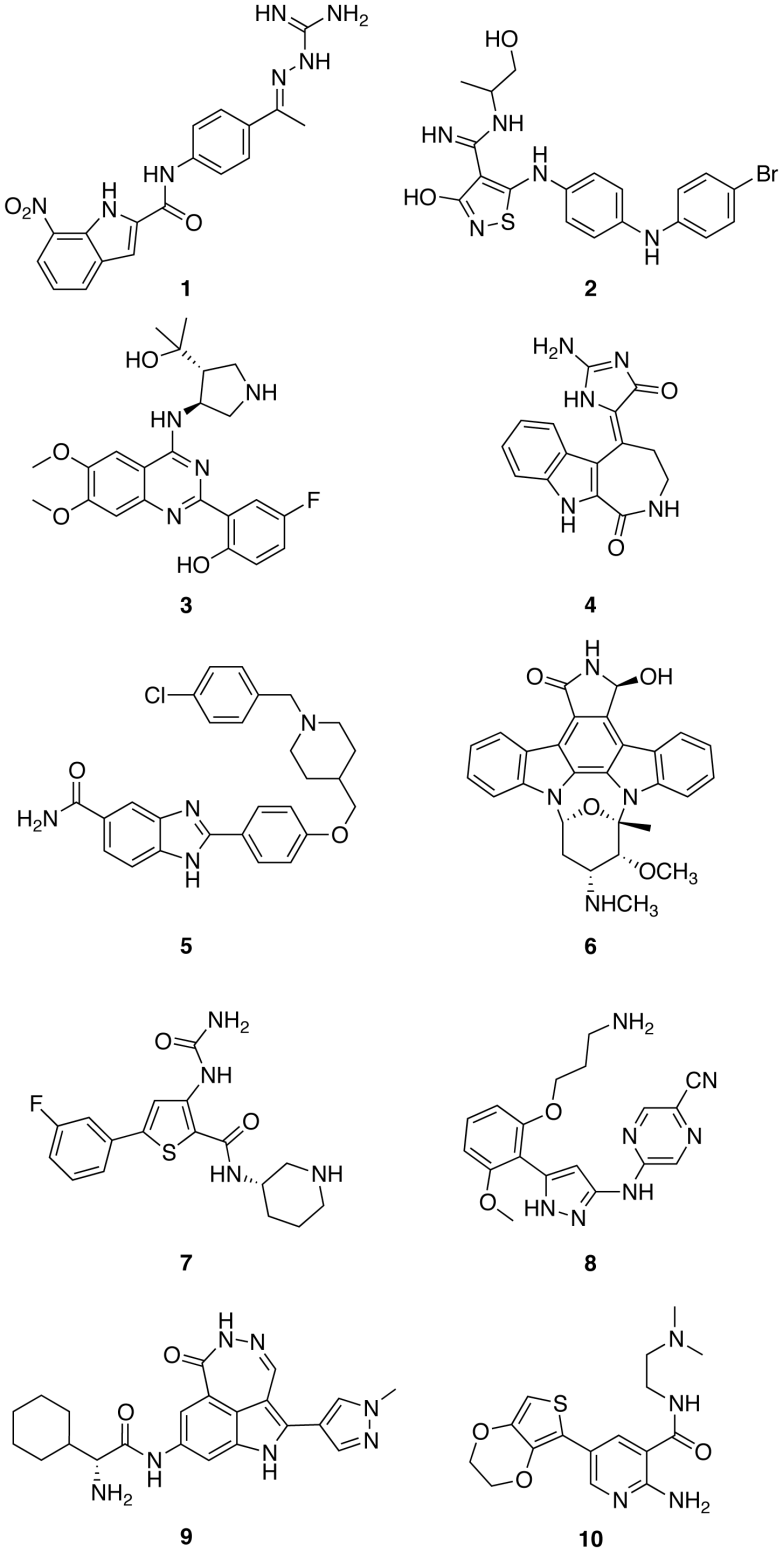
Chemical structures of published CHK2 inhibitors. **1**, The guanylhydrazone PV1019; **2**, the isothiazole carboxamidine VRX0466617; **3**, the 2-(quinazolin-2-yl-phenol inhibitor CCT241533; **4**, the indoloazepine derivative of hymenialdisine; **5**, a 2-arylbenzimidazole-5-carboxamide; **6**, the staurosporine analog UCN-01; the dual CHK1/CHK2 inhibitors **7**, AZD7762; **8**, LY2606368; **9**, PF-00477736; and **10**, a 2-aminopyridine inhibitor CHK2 inhibitor.

The therapeutic value of CHK2 inhibition is still unclear, but selective CHK2 inhibitors could be potentially beneficial in a variety of contexts. In several cancer cell lines, CHK2 is highly activated, suggesting a crucial role in survival. Therefore, inhibition of CHK2 could have the potential to exert an anti-cancer effect through disruption of DNA-repair pathways pivotal for the survival of cancer cells with high levels of activated CHK2 [1], [4], [16]. Indeed, siRNA knockdown of CHK2 and selective CHK2 inhibition with the small molecule inhibitor PV1019 (**1**, Figure 1) both resulted in an antiproliferative effect in cancer cell lines [17].

However, CHK2 inhibition is mostly being explored in the context of DNA damaging cancer therapies, such as genotoxic agents and ionising radiation. In normal cells, p53-mediated apoptosis is one of the causes of cell death in response to double strand DNA breaks caused by ionising radiation or cytotoxic chemotherapy [18]. Because approximately half of all cancers have a defective p53 tumour suppression function [19], CHK2 inhibition could selectively reduce p53-mediated apoptosis in normal tissue and therefore mitigate the side-effects of such therapies in patients with this profile [4], [20]. Experiments with four small molecule CHK2 inhibitors of different chemical classes have demonstrated such a radioprotective effect in isolated mouse thymocytes and human T-cells [17], [21], [22], [23]. In addition, it has been shown that Chk2^−/−^ transgenic mice are resistant to apoptosis after exposure to ionising radiation [3], [24] and, in contrast to p53-deficient mice, no increased tumorigenesis has been observed in these CHK2-deficient mice.

On the other hand, it has been proposed that CHK2 inhibition in p53-deficient tumor cells could sensitise the cells to DNA damaging therapies through abrogation of the G2 checkpoint [4], [25]. The validity of this hypothesis remains unclear, because although both CHK2 siRNA knock-down experiments and CHK2 inhibition by the small molecule inhibitor PV1019 showed potentiation of the cytotoxicity of topotecan and campothecan in ovarian cancer cell lines [17], no such effects have been observed with the inhibitors VRX0466617 (**2**) [22] and CCT241533 (**3**) [23], [26] (Figure 1) in combination with genotoxic agents. However, it was recently demonstrated that the potent and selective CHK2 inhibitor **3** potentiates the cytotoxicity of poly(ADP-ribose) polymerase (PARP) inhibitors such as AG14447 and olaparib, potentially providing new therapeutic options for targeted cancer therapy [26].

To date, several ATP-competitive CHK2 inhibitors have been discovered including the guanylhydrazones such as PV1019 (**1**) [17], [27], the isothiazole carboxamidines exemplified by VRX0466617 (**2**) [22], [28], an indoloazepine derivative of hymenialdisine (**4**) [29], [30] and the 2-arylbenzimidazole-5-carboxamides (**5**) [21], [31] (Figure 1). In addition, several dual checkpoint kinase 1 (CHK1)/CHK2 inhibitors with a high affinity for CHK2 have been reported, such as the staurosporine analogue UCN-01 (**6**) [32], [33], the thiophene-2-carboxamide AZD7762 (**7**) [34], [35], the *N*-(1*H*-pyrazol-5-yl)pyrazin-2-amine LY2606368 (**8**) [36], [37], the 1*H*- [1], [2]diazepino[4,5,6-*cd*]indol-6(5*H*)-one PF-00477736 (**9**) [38] and XL-844 (structure undisclosed) [39] (Figure 1). Furthermore, we have recently reported two different series of potent CHK2 inhibitors, the 3,5-disubstituted-2-aminopyridines such as (**10**) [40] and the 2-(quinazolin-2-yl)phenols which include the potent and selective CHK2 inhibitor, **3**, mentioned above [23], [26]. Both series originated from biochemical screening of focussed libraries, but in order to generate additional medicinal chemistry starting points we embarked on parallel fragment screening of CHK2.

Fragment-based drug discovery (FBDD), which in the last 10 years has become established as an attractive approach in drug discovery, involves the screening of a relatively small library, typically of 500 to 2000 compounds of low complexity and low molecular weight [41], [42], [43], [44]. Although fragments tend to bind in a highly ligand-efficient manner, their binding is often weak and fragment screening usually relies on sensitive biophysical technologies such as nuclear magnetic resonance (NMR), X-ray crystallography, surface plasmon resonance (SPR) or differential scanning fluorimetry (DSF)/thermal shift assays. However, fragment screening using high-concentration biochemical assays is increasingly being employed [45], [46], [47].

In this article, we describe the screening of our fragment library against CHK2 using a combination of a high-concentration Amplified Luminescent Proximity Homogeneous Assay Screen (AlphaScreen™) kinase assay and a thermal shift assay. A detailed comparison of the AlphaScreen™ and thermal shift screening data revealed that this combination of technologies can help prioritise the most promising fragments by the efficient identification of false positives from each individual screen. In addition, we present the protein-ligand crystal structures of nine fragment hits, all of which bind to the hinge in the CHK2 ATP-binding site. We show that with a focussed similarity search against a moderately sized library of 71,000 lead-like compounds, we were able to identify inhibitors with improved potency with respect to their different parent fragment hits, whilst maintaining ligand efficiency. The crystal structure of a quinoxaline-based follow-up compound shows it extending deeply into a previously unexplored hydrophobic pocket above the hinge region, an area that is inaccessible in CHK1 due to its larger gatekeeper +2 residue and therefore could offer a way to enhance CHK2/CHK1 selectivity in future CHK2 inhibitors.

## Results and Discussion

### The ICR Fragment Library

In order to compile an in-house fragment library, we identified 14,533 compounds from vendor libraries that passed criteria based on the Rule-of-Three [48] outlined in the materials and methods section and were available in quantities of 50 mg or greater. However, in keeping with the experience of others [49] and based on our own experience in the template screening of checkpoint kinase 1 [47], we did not adhere completely to the Rule-of-Three. In particular, we applied a maximum molecular weight filter of 300 Da with an additional 20 Da for compounds containing specific groups (F, Cl, SO_2_), in order to capture compounds with sufficient size and functionality to allow reliable detection in a high-concentration biochemical assay and to provide synthetic handles for further optimization. Based on diverse subset selection [50] and removal of compounds with undesirable structural moieties, a final selection of 1,869 fragments was purchased. This initial fragment library was screened to identify inhibitors of CHK2, which additionally allowed us to assess the performance of this first iteration of the library. In parallel, we conducted an analysis of fragment solubility and integrity using nephelometry and LC-MS, respectively, as fragment screening and subsequent crystallographic analysis usually requires experiments at high fragment concentrations [51].

### High Concentration Biochemical Fragment Screening

To identify fragments binding in the ATP-binding-site of CHK2, we screened the in-house fragment library consisting of 1869 compounds, as described above, against full-length CHK2 using an AlphaScreen™ kinase assay, in which inhibition of full-length CHK2 was measured by a reduction in the phosphorylation of a CDC25C peptide (Figure S1). Because of the generally weak affinity of fragments, the assay was carried out at a high compound concentration (300 µM). All fragments were assayed in triplicate and fragments with a percentage inhibition greater than 50% in two out of three measurements were defined as hits, yielding 45 initial hits in total, a hit rate of 2.4%. All 45 hits were confirmed by re-assaying them under the same conditions. To eliminate potential false positives due to aggregation of poorly soluble fragments, or owing to interference with the AlphaScreen™ signal, the hits were assayed by including 0.01% *(v/v)* Triton™ X-100 in the assay buffer, and in the presence of phosphorylated rather than unphosphorylated peptide substrate, respectively. The average robust Z’ for the confirmation assays was 0.9. No aggregating fragments were detected, but 17 out of the 45 hits from the primary screen were found to interfere with the AlphaScreen™ assay, with an inhibition of more than 20% of the AlphaScreen™ signal. A further eight fragments showed some interference, but this did not account for all of the inhibition seen. For the twenty fragments that showed no interference, a microfluidic mobility shift assay (see materials and methods) was used to determine the IC_50_ values, which ranged between 2.7 and 944 µM. The final confirmed hit rate for the assay was 1.1%.

### Thermal Shift Assay

In parallel, we screened the fragment library against the kinase domain of CHK2 (CHK2-KD, residues 210-531) using a thermal shift assay. In a thermal shift assay, the folding stability of a target protein is measured by its thermally-induced unfolding [52]. An increase in melting temperature of a protein in the presence of a ligand is used to identify ligand binding, assuming that the bound ligand stabilizes the target protein and therefore increases the energy required for its thermal unfolding. The thermal unfolding of CHK2-KD was measured using the fluorescent dye SYPRO® Orange™, which is sensitive to its environment and preferentially binds to hydrophobic patches that are typically exposed upon protein unfolding. To identify the hit threshold, we calculated the standard deviation (SD) of the melting temperature of CHK2 in the presence of a ligand (*T*_m, ligand_) for each plate. Ligands with a *T*_m, ligand_ value of more than 2 standard deviations above the mean *T*_m, ligand_ for each plate in at least one of the duplicates were defined as hits. We calculated the mean change in melting temperature from duplicate measurements (Δ*T*_m, ligand_) by subtracting the mean melting temperature of six reference samples of protein without ligand (*T*_m, 0_) from the melting temperature of CHK2 samples with ligand (*T*_m,__ligand_). This hit criterion resulted in 63 thermal shift hits with Δ*T*_m, ligand_ varying between 0.9 and 7.0°C, representing a hit rate of 3.4%.

### Comparison of AlphaScreen™ and Thermal Shift Results

**Figure 2:**
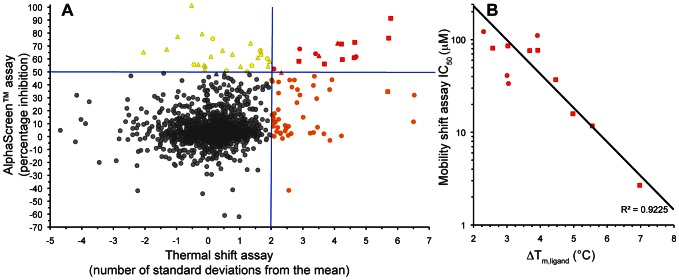
Fragment screening data from biochemical and thermal shift assays. (A) Comparison showing the primary AlphaScreen™ data plotted along the vertical axis as percentage inhibition, and the thermal shift data plotted along the horizontal axis as the number of standard deviations from the mean *T*_m, ligand_ for each plate. The hit threshold for the AlphaScreen™ is indicated by the horizontal line, the threshold for hits in the thermal shift assay by the vertical line. Hits in AlphaScreen™ and thermal shift are displayed in yellow and orange respectively. Mutual hits are shown in red. Fragments that are inactive in both assays are coloured grey. Each fragment is shown as an individual point. Fragments showing interference in the AlphaScreen™ are indicated as triangles. Fragments confirmed in crystallography are shown as squares. (B) Comparison of the IC_50_ and Δ*T*_m, ligand_ values for the screening hits. The mobility shift IC_50_ values are plotted on the vertical axis against the mean Δ*T*_m, ligand_ for each of the non-interfering mutual hits from the primary screen. The figures were generated in Microsoft Excel.

**Figure 3:**
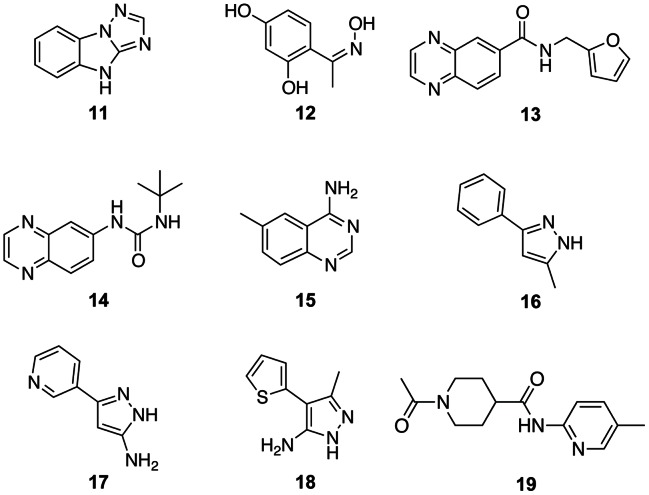
Chemical structures of the nine fragment hits confirmed in crystallography. The benzimidazotriazole compound **11** is the most active hit in both the AlphaScreen™ and thermal shift assay. Compound **12** is a resorcinol, compounds **13** and **14** both contain a quinoxaline scaffold, compound **15** is an aminoquinazoline, compounds **16**, **17** and **18** are fragments containing a pyrazole moiety, and compound **19** is a pyridine.

Comparing the primary AlphaScreen™ and thermal shift results shows that the data can be grouped into four broad categories (Figure 2A). The first category (Figure 2A, shown in red) comprises 14 mutual hits in the AlphaScreen™ and thermal shift assays, 12 of which could be confirmed by IC_50_ determination in the mobility shift assay. We observed a very good correlation of the IC_50_ and Δ*T*_m, ligand_ values (Figure 2B). None of these compounds was flagged as insoluble by nephelometry. Compound **11** was identified as the top-ranking hit in both assays with a mean IC_50_ of 2.7±0.2 µM and a Δ*T*_m, ligand_ of 7.0±0.8°C (Figure 3, Table 1). Two fragments in this category showed interference in the counter screen and were removed from the hit list. The final hit rate from combining both screens followed by the interference assay was therefore 0.64%.

**Table 1:** Crystallographically confirmed fragment hits.

Crystallographicallyvalidated hits	AlphaScreen™(percentage inhibition)^a^	Mobility shift IC_50_ (µM)^b^	*T*_m, ligand_ (standard deviations from theplate mean)^c^	Δ*T*_m, ligand_ (°C)^d^	Ligand Efficiency(kcal mol^−1^ HA^−1^)^e^
**11**	91.4±0.4	2.7±0.2	5.8	7.0±0.8	0.64
**12**	58.2±1.7	85.6±7.9	2.9	3.0^f^	0.47
**13**	76.1±1.1	11.7±2.0	5.7	5.6±0.3	0.36
**14**	59.5±1.9	36.9±9.0	4.3	4.5±0.02	0.34
**15**	72.8±6.7	15.9±1.1	4.6	5.0±0.6	0.55
**16**	56.1±1.0	81.1±24.5	3.7	2.6±1.7	0.47
**17**	71.5±25.5	76.2±3.3	4.2	3.7±0.9	0.47
**18**	60.9±2.1	76.8±1.4	4.6	3.9±1.3	0.47
**19**	34.7±0.93	227.7±22.2	5.7	4.0±0.6	0.26

a Primary screening data, expressed as the mean ± standard deviation for triplicate determinations at a compound concentration of 300 µM. The positive control compound **27** (See Figure S4 for details) gave a percentage inhibition of 62.7±2.2 at a concentration of 10 µM.

b Mean ± standard deviations for triplicate measurements. The positive control compound **28** (See Figure S4 for details) gave an IC_50_ value of 0.37±0.1 µM.

c The highest value of two independent measurements.

d Mean Δ*T*_m, ligand_ of two independent measurements. The positive control ATP gave a Δ*T*_m, ligand_ of 7.2°C at a concentration of 2 mM.

e Ligand efficiencies were calculated using the mean mobility shift assay IC_50_ values [84].

f Value from a single measurement.

The second category (displayed in yellow in Figure 2A) consists of fragments classed as actives in the biochemical screen, but with a Δ*T*_m, ligand_ below the hit threshold in the thermal shift assay. Analysis of the interference data shows that the majority of these fragments display interference with the AlphaScreen™ signal, and eight were flagged as insoluble by nephelometry.

The third category (shown in orange in Figure 2A) contains the fragments classed as hits in the thermal shift assay, but as inactives in the AlphaScreen™ kinase assay. Therefore this category is likely to include the fragments that bind to the CHK2 kinase domain, but as they do not affect the activity of the enzyme, they may bind non-specifically, or to sites other than the ATP-binding site. However, so far we have not obtained evidence of fragments binding in such second sites. Notably, this category contains three compounds that generated *T*_m, ligand_ values of more than 5 standard deviations from the mean, which is equal or better than those of the best hits in the first category. These compounds were soluble as analyzed by nephelometry; however, consistent with the initial screening data, follow-up experiments revealed IC_50_ values considerably higher than those of the mutual hits in category 1 (Figure S2). In addition, two of the three compounds failed to yield crystals in co-crystallization experiments with CHK2-KD and were not further progressed. Out of the 49 fragments in this category, 13 were determined to be insoluble by nephelometry.

The fourth and largest category includes all compounds falling below the hit thresholds in both assays, thus comprising the inactives in both screens (shown in grey in Figure 2A).

### Structural Characterisation of the Fragment Hits

**Figure 4:**
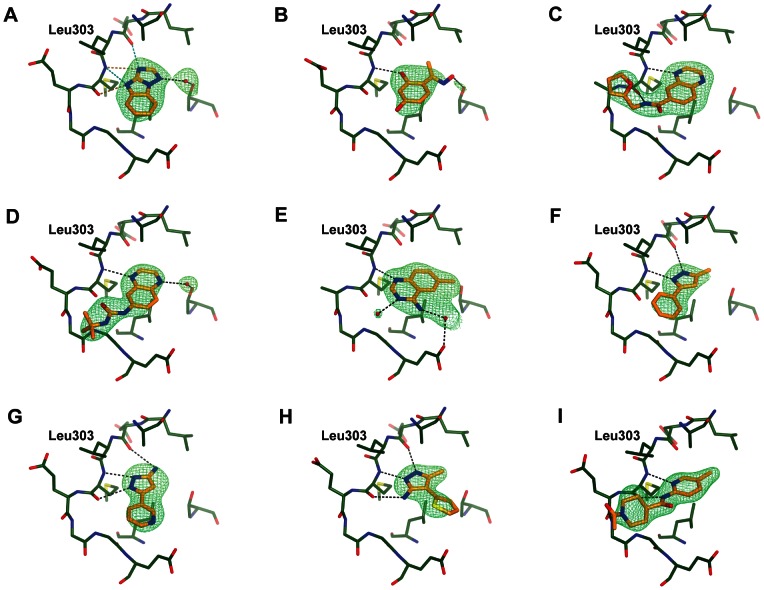
Crystal structures of CHK2 in complex with fragment hits. (A) compound **11**; (B) compound **12**; (C) compound **13**; (D) compound **14**; (E) compound **15**; (F) compound **16**; (G) compound **17**; (H) compound **18**; (I) compound **19**. Fragments are shown in cylinder representation with orange carbon atoms, and the Fo-Fc electron density omit map is shown in green and contoured at 3σ. All structural figures were generated using the graphics program CCP4MG [85].

We have obtained protein-ligand structures of nine fragment hits by co-crystallizing them with the CHK2-KD protein also used in the thermal shift assay (Figure 3). Eight fragments belong to the category of mutual hits identified in both the AlphaScreen™ and the thermal shift assays. They comprise the benzimidazotriazole **11**, the resorcinol **12**, two quinoxalines (**13** and **14**), the amino-quinazoline **15**, and three pyrazole-containing fragments (**16**, **17** and **18**). Compound **19** was the only fragment of the three fragments from the third category (those with a strong thermal shift but minimal biochemical activity) that yielded a crystal structure. All nine fragment hits bind to the hinge region in the CHK2 ATP-binding pocket (Figure 4), although the relatively poor ligand efficiency of compound **19** makes it an unattractive fragment to follow-up (Table 1); therefore, we removed it from further analyses. Although the eight mutual fragment hits all bind to CHK2-KD with one or more of the canonical hydrogen-bond interactions (Figure 4), there are some interesting differences in the way they bind to the hinge.

The binding of compound **11**, which is the top-ranking hit in both the AlphaScreen™ kinase and thermal shift assays, is complicated as it can adopt different tautomers. Thus, compound **11** interacts with the hinge either through hydrogen bonds with the backbone carbonyl of Glu302 and the backbone amide of Met304, or *via* hydrogen bonds of both the backbone amide and carbonyl groups of Met304. Unfortunately, the structural data do not allow discrimination between these possibilities and it may even be the case that a mixture of tautomers is present in the crystal. In addition to the interactions with the hinge region, compound **11** interacts *via* a mediating water molecule with the side-chain hydroxyl of Thr367, located at the start of the activation loop just before the DFG motif.

By contrast, the resorcinol compound **12** forms only a single hydrogen bond with the hinge through one of its hydroxyl groups and the backbone amide of Met304. An additional weak interaction is made *via* a CHO-interaction with the backbone carbonyl of Met304. Furthermore, compound **12** interacts directly with the side chain of Thr367, instead of *via* a mediating water molecule as seen in the compound **11**-bound structure.

**Figure 5:**
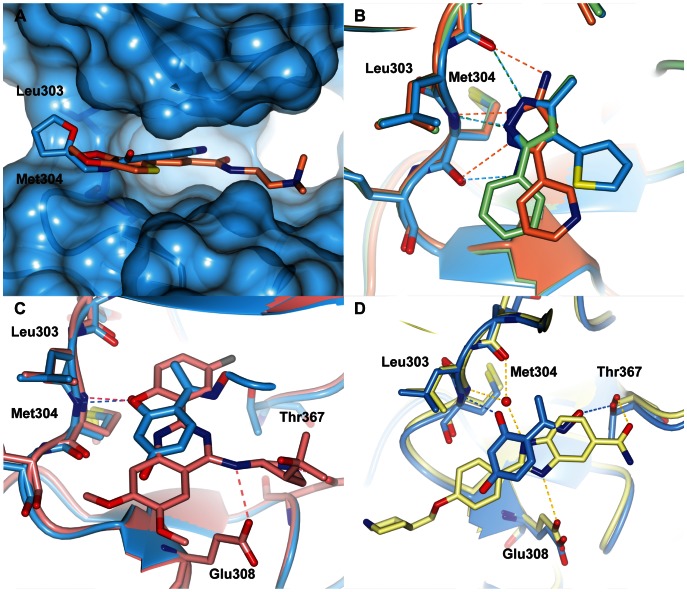
Fragments map interaction hotspots exploited by known CHK2 inhibitors. (A) Superposition of compound **13** (light blue) with the 2-aminopyridine inhibitor, compound **10** (orange, PDB code 2WTJ) showing both interacting with the CHK2 surface defined by Leu303 and Met304, an area suggested to be important for hydrophobic interactions. (B) Superposition of the three pyrazole fragments compound **16** (green), **17** (orange) and **18** (light-blue) showing the differences in binding. (C) Superposition of the resorcinol compound **12** (light-blue) and the 2-(quinazolin-2-yl)phenol inhibitor, compound **3** (pink, PDB code 2XBJ). (D) Comparison of compound **12** (light-blue) with the benzimidazole inhibitor **5** (yellow, PDB code 4A9U). Note that the chlorobenzyl group of **5** is not modeled in the crystal structure and has not been included in the PDB coordinates.

The two quinoxaline fragments, compounds **13** and **14**, bind in a very similar way, with a hydrogen bond to the hinge between one of the ring nitrogen atoms and the amide nitrogen of Met304 and a CHO-interaction with the backbone carbonyl of Glu302. Compound **13** forms an additional hydrogen bond with the protein between its amide N2 atom and the carbonyl group of Met304. Furthermore, the furan ring in compound **13** binds to the surface defined by Leu303 and Met304, an area associated with productive hydrophobic interactions and probed in the previously-described 2-aminopyridine CHK2 inhibitors [40], such as 2-amino-5-(2,3-dihydrothieno[3,4-*b*] [1], [4]dioxin-5-yl)-*N*-(2-(dimethylamino)ethyl)nicotinamide (**10**), shown in Figure 1 and 5A. Similar to compound **11**, compound **14** also interacts with Thr367 *via* a mediating water molecule, which is not present in the compound **13**-bound structure. The electron density of compound **14** indicates that the oxygen atom of its urea moiety points towards the carbonyl group of Met304. This is surprising, as it seems an unfavorable interaction; however, it may account for the slight difference in potency between the two quinoxaline fragments.

The amino-quinazoline compound **15** also interacts *via* one of its ring nitrogen atoms with the amide group of Met304, but in addition its amino-group forms an interaction with Glu308 *via* a mediating water molecule.

The identification of the three pyrazole fragments was reassuring, because the pyrazole moiety is well precedented as a hinge-binding motif in kinase inhibitors [53], [54]. Interestingly, although the pyrazole group in all three hits is the hinge-binding motif and occupies the same space, the three fragments bind in a different manner due to the substitution pattern of each compound (Figure 5B). Compound **16** binds along the hinge with the pyrazole group forming two hydrogen bond interactions with the backbone carbonyl and amide groups of Glu302 and Met304 respectively. Compound **17** also binds along the hinge, but is offset by approximately 26° compared to compound **16**. In this fragment the interaction with the backbone carbonyl of Glu302 is made by the amino-substitution on the pyrazole ring and the pyrazole group itself interacts with both the amide and carbonyl groups of Met304, explaining the rotation of the fragment compared to compound **16**. The third pyrazole fragment, compound **18**, binds in an almost orthogonal way with respect to compound **16** as a result of the thiophene substitution on the pyrazole 4-position compared to the phenyl substitution on the pyrazole 3-position in compound **16**. However, a detailed comparison shows that the pyrazole moieties of the two fragments overlay almost perfectly and make the same interactions with the hinge. In addition, in both compounds the 5-methyl groups superimpose very well and bind in a small hydrophobic pocket near the gatekeeper Leu301.

Comparison of these fragments with known CHK2 inhibitors shows that the fragments are able to map several interaction hot spots in the CHK2 ATP-site. Not surprisingly, the different possible interactions with the hinge are represented in the different fragments. However, it is interesting to note that the 2-(quinazolin-2-yl)phenol CHK2 inhibitors, including **3**, do not bind to the hinge through their quinazoline scaffold as observed for fragment **15**. Instead, they interact with the hinge *via* a hydrogen bond between the phenolic oxygen and the backbone amide group of Met304, similar to the hydroxyl-hinge interaction of the resorcinol fragment **12** (Figure 5C) [23]. Intriguingly, although their respective hydroxyl groups occupy the same space, which is also the location of the mediating water molecule in the NSC109555- [55] and PV1019-bound [17] structures, the aromatic parts of compound **12** and the phenol-moiety in the 2-(quinazolin-2-yl)phenol inhibitors do not superimpose (Figure 5C). In addition, the water-mediated interaction of compound **15** with Glu308 was also observed in compound **10** from the 2-aminopyridine CHK2 inhibitors and exploited as a direct-protein inhibitor interaction in the 2-(quinazolin-2-yl)phenol CHK2 inhibitor series (Figure 5C). Furthermore, the interaction with the side chain of Thr367 observed with compounds **11** and **14** (water-mediated) and compound **12** (direct) is also found in a series of potent benzimidazole-based CHK2 inhibitors, such as 2-(4-((1-benzylpiperidin-4-yl)methoxy)phenyl)-1*H*-benzo[*d*]imidazole-5-carboxamide [56] (**5**, Figure 5D). Finally, the surface of Leu303 and Met304 binding the furan group in compound **13** has been postulated as an area for hydrophobic interactions [40], but to date has not been explored in the rational design of CHK2 inhibitors.

### Fragment Hit Expansion

**Figure 6:**
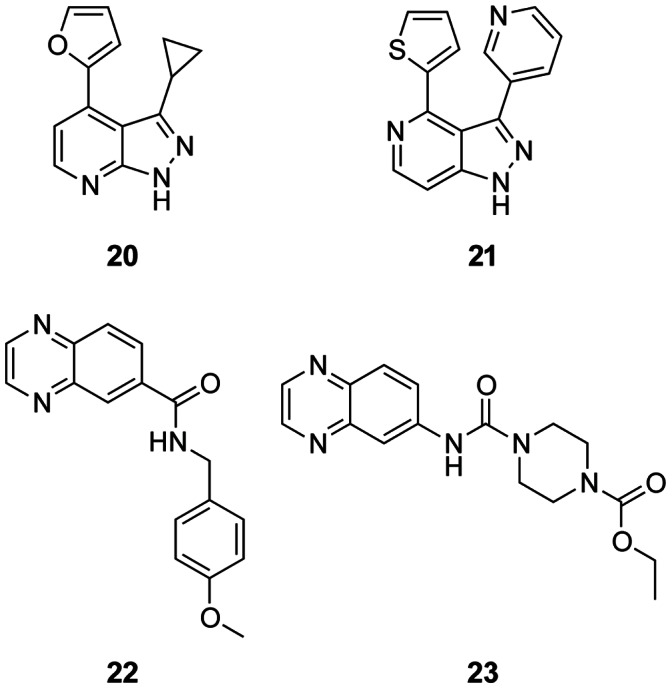
Chemical Structures of the four follow-up compounds selected for crystallography. Compound **20** and **21** both originate from the pyrazole fragments **17** and **18** and compound **22** and **23** are both quinoxalines relating to compound **13**.

For further confirmation and initial elaboration of the identified chemotypes, a similarity search was carried out using an in-house HTS library, comprising approximately 71,000 compounds with lead-like physicochemical properties. For the search, we selected the twenty confirmed AlphaScreen™ hits and a further twenty hits from the fragments with the largest thermal shift in screening. The resulting set of 40 fragments included the eight mutual and structurally confirmed fragment hits and the similarity search was set up to find the ten most similar compounds for each fragment. Compounds were chosen for further testing based on their similarity score, similar connectivity as the parent fragment, a molecular weight larger than that of the parent fragment, and visual inspection. This yielded 132 compounds in total, for which the percentage inhibition was determined in the mobility shift assay at three concentrations. Nineteen compounds were selected for IC_50_ determination based on the percentage inhibition data and on chemotype. Subsequently, four of these compounds (**20**–**23**) were selected for co-crystallization experiments (Figure 6, Table 2).

**Table 2:** Selected follow-up compounds from similarity search.

Compounds selected for crystallography	Mobility shift IC_50_ (µM)^a^	Ligand Efficiency(kcal mol^−1^ HA^−1^)^b^	IC_50_ of parent fragment(s) (µM)
**20**	7.2±2.9	0.42	76.2
**21**	21.3±2.1	0.32	76.8
**22**	3.6±1.2	0.34	11.7/36.9
**23**	14.2±8.6	0.28	11.7/36.9

a The IC_50_ values are expressed as mean ± standard deviation from triplicate measurements. The positive control compound **28** (See Figure S4 for details) gave an IC_50_ value of 0.30±0.1 µM.

b Ligand efficiencies were calculated using the mean mobility shift assay IC_50_ values.

**Figure 7:**
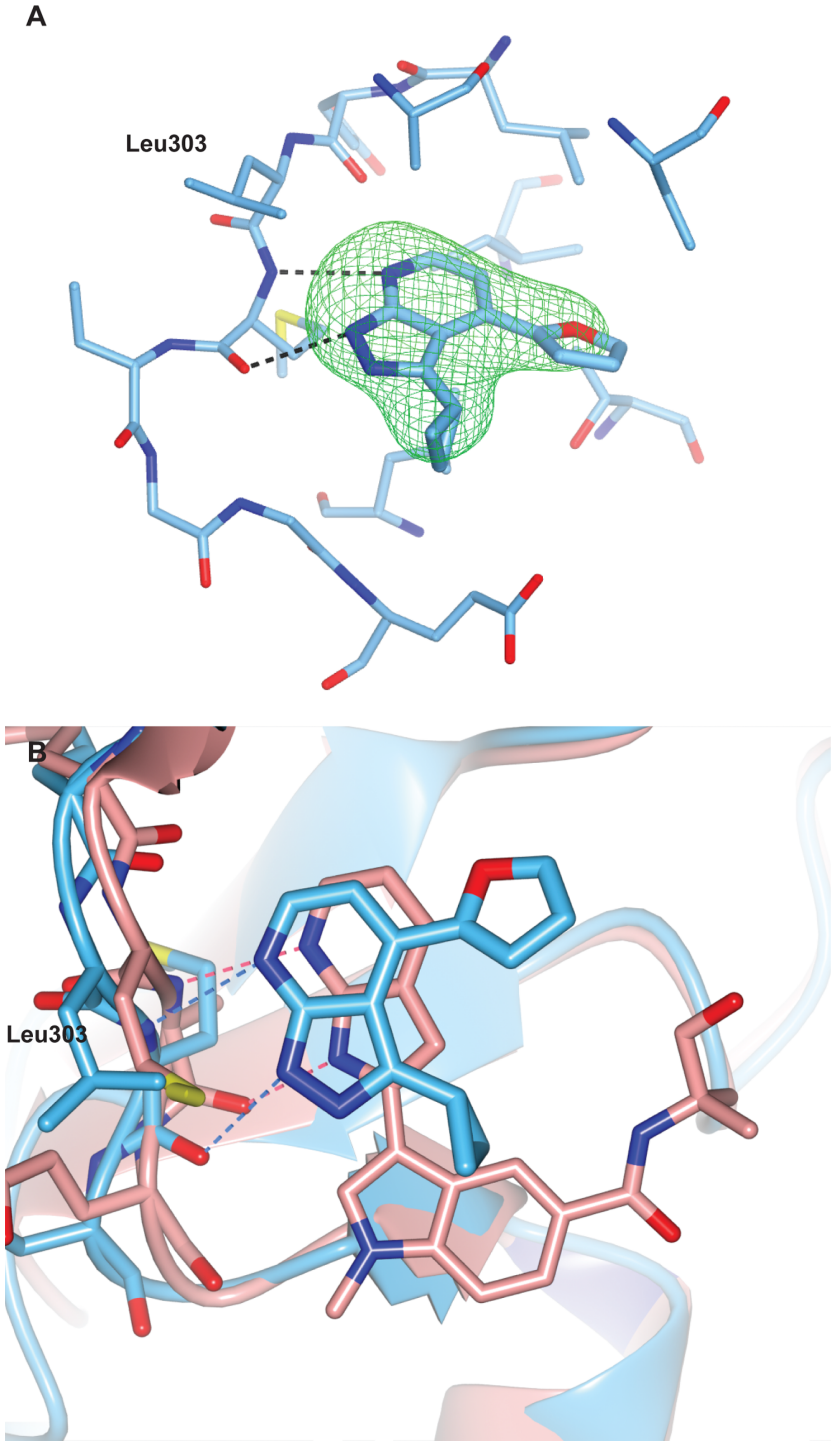
The binding mode of the pyrazolopyridine compound 20. (A) Crystal structure of CHK2 in complex with compound **20**. The compound is shown in cylinder representation with light-blue carbon atoms, and the Fo-Fc electron density omit map is shown in green and contoured at 3σ. (B) Superposition of compound **20** (light-blue) and the spleen tyrosine kinase inhibitor **24** (pink, PDB ID code 3FQH) [58] showing they bind in a similar way to the hinge gatekeeper +3 residue.

Compounds **20** and **21** are both more potent than their parent fragments **17** and **18**. We were able to obtain a crystal structure of CHK2 complexed with the pyrazolopyridine compound **20**, the more potent of the two, but not with compound **21**. Compound **20** binds in a different way to the hinge compared to its parent pyrazole fragment. Interestingly, it forms hydrogen bonds with the backbone amide and carbonyl groups of Met304, the gatekeeper +3 residue *via* its N7 and N1 atoms, respectively, thus positioning the pyrazole ring towards the solvent-exposed region of the ATP binding site (Figure 7A). This is a different binding mode than observed, for example, in a series of pyrazolopyridine inhibitors of CHK1 [47], which interact with the hinge region with the pyrazole facing the gatekeeper and forming hydrogen bonds with the backbone carbonyl of the gatekeeper +1 residue and the backbone amide of the gatekeeper +3 residue. A search for protein-ligand structures exemplifying the binding mode of the pyrazolopyridine scaffold of compound **20** did not yield any results; however, I*κ*B kinase subunit *β* (IKK2) inhibitors containing a 7-azaindole scaffold have been postulated to bind in analogous pattern to the kinase hinge [57]. Moreover, a crystal structure of a 7-azaindole containing inhibitor of spleen tyrosine kinase (Syk), *N*-(1-hydroxy-2-methylpropan-2-yl)-1-methyl-3-(1*H*-pyrrolo[2,3-*b*]pyridin-2-yl)-1*H*-indole-5-carboxamide (**24**), shows the 7-azaindole binding in this manner (Figures 7B, S3) [58].

**Figure 8:**
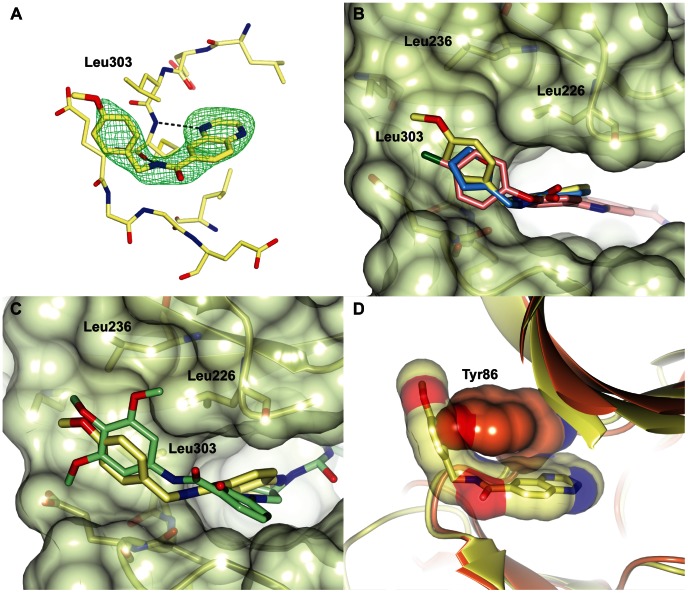
The binding mode of the quinoxaline compound 22. (A) Crystal structure of CHK2 in complex with compound **22**. The compound is shown in cylinder representation with yellow carbon atoms, and the Fo-Fc electron density omit map is shown in green and contoured at 3σ. (B) Superposition of the quinoxaline fragment compound **13** (light-blue), compound **22** (yellow) and the arylbenzimidazole CHK2 inhibitor **26** (pink, PDB ID code 4A9R), showing the fragments bind in a nearly identical manner with their respective furan and *p*-methoxyphenyl group binding in a hydrophobic pocket above the hinge, which is also accessed by the chlorophenyl group of the CHK2 inhibitor **26**. CHK2 is shown in a semitransparent surface representation and the location of Leu226, Leu236 and Leu303 are indicated. (C) Superposition of compound **22** (yellow) and the JNK3 inhibitor **25** (light-green), showing that their respective *p*-methoxyphenyl and trimethoxyphenyl groups bind in the hydrophobic pocket above the hinge. (D) Superposition of the compound **22**-bound CHK2 structure in yellow and the apo-structure of CHK1 (PDB ID code 1IA8) in orange, showing the clash of compound **22** with Tyr86, the gatekeeper +2 residue in CHK1.

Compounds **22** and **23** are both quinoxalines and are related to the parent quinoxaline fragment compounds **13** and **14**, respectively. Compound **22** is the most potent of the twenty compounds tested and is modestly more potent than its parent fragment **13**. The crystal structure of compound **22** bound to CHK2 shows that its binding mode is nearly identical to that of compound **13**. Both bind in the CHK2 ATP-binding site and interact with the hinge in the same manner and their respective furan and *p*-methoxyphenyl groups both extend into a previously unexplored hydrophobic crevice defined by Leu303, Met304, Glu305, Leu226, Leu236 and Lys245 (Figure 8A). The main difference between the two compounds is the orientation of the *p*-methoxyphenyl group of compound **22** with respect to the furan ring of compound **13** (Figure 8B). The *p*-methoxyphenyl moiety of compound **22** packs against Leu303, the gatekeeper +2 residue in CHK2, in a similar manner to the interaction of the trimethoxyphenyl-groups of the indazole and aminopyrazole-based c-Jun N-terminal kinase 3 (JNK3) inhibitors SR-3737 and SR-3451 (**25**) [59] with Leu148, the gatekeeper +2 residue in JNK3 (Figures 8C, S3).

A superposition of the compound **22**-bound CHK2 structure with the apo-structure of CHK1 [60] shows that compound **22** would clash with Tyr86, the gatekeeper +2 residue in CHK1 (Figure 8D), suggesting that exploiting this pocket could enhance the selectivity of the next generation of CHK2 inhibitors. Intriguingly, we recently showed that the chlorophenyl group of one of the early CHK2 selective arylbenzimidazole inhibitors **26** (Figure S3) binds in this region [56], although in a slightly less extended manner as compared to the *p*-methoxyphenyl group of compound **22** (Figure 8B). However, it is very difficult to assess the contribution to selectivity of binding in this pocket using the inhibitor **26**, because its CHK2 selectivity is most likely dominated by the unusual binding of the benzamidazole scaffold to the hinge through a mediating water molecule. Nevertheless, the crystallographic data, combined with the fact that both the fragment **13** and its follow-up compound **22** are inactive in a CHK1 mobility shift assay (IC_50_>200 µM for both compounds), make it enticing to postulate that this pocket could potentially be exploited to enhance both affinity and selectivity of future CHK2 inhibitors.

### Conclusions

The use of orthogonal techniques in fragment screening and a subsequent focus on the common hits is seen as a key to success in fragment screening [61]. However, comparisons between SPR, NMR and DSF/thermal shift assays [51], high concentration biochemical screening versus NMR [51], NMR versus SPR [62], and SPR versus a high-concentration mobility shift assay [63] revealed a varying degree of correlation between hits discovered using different screening methods. Here we have used the biochemical AlphaScreen™ kinase assay and biophysical thermal shift assay to screen a fragment library for inhibitors of CHK2 and found a good correlation between the hits identified by each method. Importantly, the orthogonal use of these two assays allowed us to quickly focus on the most promising fragment hits, and would also be very helpful in reducing the false-positive hit rate in cases where an interference assay is not available or practical. We have identified a number of chemically different ligand-efficient fragment hits for CHK2 and determined their binding mode using X-ray crystallography. It is of interest to point out that all structural information was obtained using co-crystallization experiments, which demonstrates that, with suitable primary screening options, the crystallographic follow-up of a fragment-based screening campaign is not necessarily reliant upon the availability of a soakable crystal system.

Although we allowed a slightly higher molecular weight cut-off than the 250 Da nowadays typically used in the design of a fragment library [42], [51], interestingly six out of the nine crystallographically confirmed fragment hits have a molecular weight below 200 Da and one has a molecular weight of 244.3 Da. With respective molecular weights of 253.3 and 261.3 Da, the other two fragment hits, compounds **13** and **19**, are only marginally larger and well below the higher molecular weight cut-off (320 Da) of our fragment library. Importantly, all hits bind to the CHK2 hinge region, including compound **19** from the category of thermal shift hits and AlphaScreen™ inactives. This hit category should include any second site binders and therefore our findings confirm the adenine subpocket as the dominant fragment-binding site.

Furthermore, we have shown that, in addition to the interactions with the hinge, these fragments exploit several of the interaction hot-spots used by advanced CHK2 inhibitors, but do so in different ways. Because no fragments were found to bind in other subpockets of the CHK2 ATP binding site, further development into potent lead molecules through fragment linking [64], [65] is not an option. However, since none of the CHK2 fragment hits is exemplified as a hinge-binding scaffold in the previously reported CHK2 inhibitors, they could be developed by merging them with existing CHK2 inhibitors. Furthermore, in keeping with the majority of advanced fragment-based kinase inhibitors, such as the B-raf inhibitor PLX4032 (Vemurafenib) [66], the PKB/Akt inhibitor AZD5363 [67], and the Aurora Janus kinase 2 inhibitor AT9283 [68], optimization using a fragment evolution/growing strategy [64] would be the most promising way to develop our CHK2 fragment hit matter into potent lead molecules with favorable physicochemical properties.

Moreover, the crystal structures of compound **13** and its follow-up compound **22** access a hydrophobic area above the hinge not previously explored in rational CHK2 inhibitor design. We speculate that this pocket could be exploited to enhance both potency and selectivity of CHK2 inhibitors. However, although compounds **13** and **22** have good ligand efficiencies (table 1 and 2), the usefulness of this pocket in CHK2 inhibitor design will need to be further investigated, starting from more potent but non-selective CHK2 inhibitors. Together the similar binding mode observed for JNK3 inhibitors, and the fact that many kinases have a phenylalanine or tyrosine residue in the gatekeeper +2 position, suggest that the area above the hinge could also be important in the design of selective ATP-competitive inhibitors for other kinases with a small gatekeeper +2 residue.

## Materials and Methods

### Design of the ICR Fragment Library

To define the fragment library parameters the following molecular weight (MW) filter was applied: 150 Da<Molecular weight (MW) <300 Da, with the MW permitted to increase by a further 20 Da for specific groups (F, Cl, SO_2_). In addition, typical Rule-of-Three-based physicochemical property filters [48] were used such as, ClogP≤3 [69], hydrogen bond acceptors ≤5 and hydrogen bond donors ≤3, a topological polar surface area (TPSA) ≤75 Å^2^ [70], and the number of rotatable bonds ≤3. Furthermore, only compounds with ten or more heavy atoms were included, compounds were allowed to have 1 to 3 rings with between 3 and 7 atoms per ring, and a maximum of 1 halogen or sulfur atom per fragment was permitted. The filters were applied using the descriptors implemented in MOE 2007.09 [50] and diverse subset selections were also carried out in MOE. Prior to purchasing, the final fragment selection was visually inspected to remove fragments with undesirable structural moieties, such as known reactive groups, Michael acceptors, and aromatic nitro groups.

Samples for solubility measurements using nephelometry and LC-MS analysis were collected from 20 mM samples in 100% DMSO and made up for analysis in 96-well plates at a final sample concentration of 500 µM and 2.5% *(v/v)* DMSO. This allowed for 80 fragments per well and 16 blank control wells for data normalization. All nephelometry experiments were carried out using a NEPHELOstar Galaxy (BMG Labtech GmbH, Ortenberg, Germany) and were performed in duplicate to minimize errors. Measurements were collected for each of the plates at a rate of 1 s per well using a gain of 80 and a beam focus of 2 mm. To account for the noise in the measurements, the reading of each well value was normalized by the average of the empty well data for each plate. The minimum of the two replicate well values was then taken as the more accurate reading. Compounds with readings of four standard deviations above the mean of all measurements were defined as insoluble.

LC-MS measurements were conducted on the same fragment plates as used for the nephelometry experiments, with the data for one replicate plate collected in positive ionization mode, while data for the other replicate plate was collected in negative ionization mode. Analytical separation was carried out at 30°C on a Merck Chromolith SpeedROD column (RP-18e, 50×4.6 mm) using a flow rate of 2 mL/min in a 4 min gradient elution with UV detection at 254 nm. The mobile phase was a mixture of methanol (solvent A) and water (solvent B), both containing 0.1% *(v/v)* formic acid. Gradient elution was as follows: 1∶9 (A/B) to 9∶1 (A/B) over 2.5 min, 9∶1 (A/B) for 1 min, and then reversion back to 1∶9 (A/B) over 0.3 min, finally 1∶9 (A/B) for 0.2 min. Positive and negative ionization was achieved on a 6520 series qToF mass spectrometer fitted with a MultiMode ionization source (Agilent, Santa Clara, USA). Fragments that failed LC-MS, or were identified as insoluble, were flagged as such in our library documentation and compound database.

### Protein Expression and Purification

The coding sequence for full-length human CHK2 (residues 1 to 543) was PCR amplified from the IMAGE clone AU20-A2 (Human Genome Mapping Project) and inserted into the pFastBac HTa vector, which encodes an *N*-terminal 6xHis-tag. Recombinant baculovirus was generated according to the Bac-to-Bac® protocols (Invitrogen, Paisley, UK). *Sf9* insect cells were grown in sf-900 II media to a cell density of around 10^6^ cells per mL, infected with 10 µL to 100 µL of virus per 10^7^ cells and harvested after 48–72 h. Cell pellets were lyzed by resuspension in lysis buffer (50 mM HEPES pH 7.4, 250 mM NaCl, 0.1% *(v/v)* NP40, 1 mM NaF, 10 mM β-glycerophosphate, 0.1 mM Na_3_VO_4_) and incubated on ice for 30 min. Following centrifugation the supernatant was diluted with 1/7 volume of 8x binding buffer (160 mM Tris pH 7.9, 4 M NaCl, 40 mM imidazole) and passed over a column containing His Bind resin (Novagen, Merck Chemicals Ltd, Nottingham, UK). The column was washed with 8 column volumes (CV) of 1x binding buffer, 3 CV of 0.5x wash buffer (20 mM Tris pH 7.9, 250 mM NaCl, 30 mM imidazole) and eluted with 3 CV of elution buffer (20 mM Tris pH 7.9, 500 mM NaCl, 500 mM imidazole). Eluted protein was dialysed against 20 mM HEPES pH 7.4, 150 mM NaCl, 1.5 mM DTT, 0.03% (*v/v*) Brij-35, 50% (*v/v*) glycerol and stored at –80°C.

The kinase domain of CHK2 (residues 210-531) was produced as a GST-fusion protein and purified as previously described [8].

### Fragment Screening Using a Biochemical Assay

Full-length CHK2 was screened against the ICR fragment library consisting of 1869 fragments using a biochemical assay based on AlphaScreen™ technology [71] in which the CHK2 kinase activity was measured by monitoring the phosphorylation of a CDC25C peptide using a phospho-specific antibody [40]. Assay conditions were similar to those described by Hilton *et al*., but all fragments were screened at a final concentration of 300 µM. Amendments to the protocol included a change in the full-length CHK2 concentration to 2 nM and a final concentration of the antibody against phosphorylated CDC25C of 0.5 nM. For a positive control, 4-(2-amino-5-(thiophen-3-yl)pyridin-3-yl)benzoic acid (**27**, Figure S4, referred to as compound 19 in Hilton *et al.*) [40] was added at a final concentration of 10 µM. The phosphorylation reaction was performed for 80 min at room temperature and stopped by the addition of 5 µL of the previously described detection buffer [40]. Plates were incubated overnight at room temperature and in the dark, and the assay endpoint was measured using an Envision™ 2103 multilabel reader (Perkin Elmer Life Sciences, Seer Green, UK). Primary screening data were analyzed in ActivityBase (IDBS, Guildford, UK). Percentage inhibition was calculated as follows: 100*(1–(S–B)/(T–B)), where S represented the counts for each compound well, B the counts in the wells containing no enzyme, and T the counts in the total activity wells. The plates were assayed in triplicate and fragments with a percentage inhibition of 50% or more in at least two out of the three measurements were defined as initial hits. Initial hits were re-assayed under the same conditions in triplicate for reconfirmation. Furthermore, all hits were tested for interference by repeating the assay in the presence of phosphorylated rather than unphosphorylated peptide substrate, and for aggregation by including 0.01% (*v/v*) Triton™ X-100 in the assay buffer.

### IC_50_ Determination Using a Mobility Shift Assay

For all fragment hits showing no interference and no aggregation, IC_50_ values were determined using a microfluidic assay that monitors the separation of a phosphorylated product from its substrate. In addition, percentage inhibition and IC_50_ values for all follow-up compounds were determined in triplicate using this assay. The assay was performed on an EZ Reader II (Caliper Life Sciences Ltd, Runcorn, UK) using separation buffer (#760367 Caliper LS) containing CR-8 (500 nM, #760278, Caliper LS). An ECHO® 550 acoustic dispenser (Labcyte Inc™, Dublin, Ireland) was used to generate duplicate eight-point dilution curves directly into 384-well low-volume polystyrene assay plates (Corning Life Sciences, New York, USA). For each compound, a 10 mM stock concentration in 100% DMSO was used. The total amount of DMSO dispensed per well was 250 nL to give a final assay concentration of 2.5% *(v/v)* DMSO and compounds in the range 0.1–200 µM. For a positive control, 2-[4-((*R*)-piperidin-3-ylamino)-quinazolin-2-yl]-phenol (**28**, Figure S4, referred to as compound 13 in Caldwell *et al.*) [23] was used in the range 0.005–10 µM.

An assay master mix consisting of 6 µL full-length CHK2 (2 nM final concentration), 2 µL peptide 10 (5-FAM-KKKVSRSGLYRSPSMPENLNRPR-COOH, 1.5 µM final concentration, #760354 Caliper LS) and 2 µL ATP (100 µM final concentration) all diluted in kinase buffer (40 mM HEPES pH 7.5, 40 mM KCl, 1 mM DTT, 2 mM MgCl_2_ and 0.02% *(v/v)* Tween20) was added to the compounds in the assay plate. The plate was sealed and centrifuged for 1 min at 1000 rpm before incubation for 1 h at room temperature. The reaction was stopped by the addition of separation buffer (760367, Caliper LS), containing 100 mM HEPES pH 7.3, 0.015% *(v/v)* Brij-35, 5% (*v/v*) DMSO, 0.1% (*v/v*) Coating reagent 3, 0.05 µM and 10 mM EDTA.

The plate was read on an EZ Reader II, using a 12-sipper chip (760137-0372R, Caliper LS) with instrument settings of −1.5 psi and 1750 ΔV. The percentage conversion of product from substrate was generated automatically and the percentage inhibition was calculated relative to blank wells (containing no enzyme and 2.5% (*v/v*) DMSO) and total wells (containing all reagents and 2.5% (*v/v*) DMSO). IC_50_ values were calculated from a four-parameter logistics fit of percentage inhibition *versus* concentration using the Studies package (from Dotmatics, Bishops Stortford, UK).

### Fragment Screening Using a Thermal Shift Assay

Thermal shift screening of the ICR fragment library against a truncated version of CHK2 comprising only the kinase domain (CHK2-KD), was carried out using an Opticon 2 RT-PCR machine (Bio-Rad, Hemel Hempstead, UK). The assay buffer consisted of 0.14 mg/mL (3.9 µM) CHK2-KD, 4.2x SYPRO® Orange protein gel stain (Sigma-Aldrich), 10 mM HEPES pH 7.5, 50 mM NaCl and 4 mM DTT in a final volume of 50 µL. All experiments were performed in white 96-well SuperPlate skirted PCR-plates (ABgene®, Thermo Scientific, Loughborough, UK). Fragments were screened at a final concentration of 2 mM in assay buffer containing a final concentration of 2% *(v/v)* DMSO and all measurements were carried out in duplicate. The well contents were mixed by centrifugation for 2 min at 500 g and pre-equilibrated for 5 min at 20°C before starting the thermal shift experiment. All melting curves were generated from 20°C to 95°C, raising the temperature in steps of 0.5°C and keeping it constant for 15 seconds at each step. The melting temperature of CHK2 in the absence of a ligand (*T*_m, 0_) was determined by averaging six reference melting curves per plate from wells containing the thermal shift assay buffer and CHK2-KD in 2% *(v/v)* DMSO. MgATP (2 mM ATP and 5 mM MgCl_2_) in the presence of 2% *(v/v)* DMSO was used as a positive control. For each experiment, the data range of the protein unfolding transition was established using the Excel-based worksheet ‘DSF Analysis’, made available by the Structural Genomics Consortium (SGC), Oxford [72], and subsequently fitted with a Boltzmann sigmoidal equation using GraphPad Prism version 5 (GraphPad Software, San Diego, California, USA, www.graphpad.com), from which the melting temperature *T*_m_ was calculated. The change in melting temperature caused by ligand binding, expressed as the mean from duplicate measurements (Δ*T*_m, ligand_), was calculated by subtracting *T*_m, 0_ from each melting temperature obtained in the presence of a ligand (*T*_m, ligand_) using the DSF-analysis spreadsheet. The hit threshold was determined by calculating the standard deviation (SD) of the melting temperatures of CHK2 in the presence of ligand (*T*_m, ligand_) for every plate. Ligands with a *T*_m, ligand_>mean(*T*_m, ligand_) +2SD in at least one of the duplicates were defined as hits.

### Similarity Search for Fragment Elaboration

A similarity search was performed against an in-house compound library, which contained 70,877 unique chemical structures with lead-like physicochemical properties. The 20 confirmed AlphaScreen™ hits and the 28 hits with the largest thermal shift were selected as probes. After removal of duplicate fragments this yielded a set of 40 parent structures, which included the eight crystallographically confirmed fragment hits. A similarity search was performed for each probe in turn and the 10 most similar compounds were selected from the compound library. The search protocol was executed in PipelinePilot 8.0 [73] using Functional-Class Fingerprints [74] with a diameter of four (FCFP_4) and similarities between the fingerprints of the compounds calculated using the Tanimoto coefficient [75].

### Crystallization and Structure Elucidation

Co-crystallization experiments with selected fragment hits were carried out based on conditions described earlier [8]. In brief, crystallization experiments were performed using the hanging- and sitting-drop vapor diffusion methods at 4°C. Crystallization drops were made by mixing 2 µL protein solution (typically 10 mg/mL CHK2-KD in buffer containing 10 mM HEPES pH 7.5, 250 mM NaCl, 10 mM DTT, 2 mM EDTA and 2 mM of a fragment hit) and 2 µL precipitant solution (0.1 M HEPES pH 7.5, 0.2 M Mg(NO_3_)_2_, 10% *(v/v)* ethylene glycol, 1 mM TCEP and 8–14% *(w/v)* PEG 3350) over 0.5 mL of the respective reservoir solution. Crystals usually grew in 2–5 days and were harvested and cryoprotected using a cryoprotectant solution containing 0.1 M HEPES NaOH pH 7.5, 0.1 M NaCl, 0.2 M Mg(NO_3_)_2_, 20% *(v/v)* ethylene glycol and 10% *(w/v)* PEG 3350 before flash-freezing in liquid nitrogen.

The datasets were collected at beamlines I02, I04 and I24 at the Diamond Light Source (Oxfordshire, UK) and integrated, merged and scaled using the programs MOSFLM (Leslie, 1992) and SCALA from the CCP4 suite (Collaborative Computational Project, Number 4, 1994 [76], see table S1 for data collection and refinement statistics), except for the data for compound **22**, which were collected on an in-house X8 PROTEUM system (Bruker AXS Ltd., Coventry, UK), and integrated, merged and scaled with PROTEUM2. All CHK2 protein-ligand structures were solved by molecular replacement using PHASER [77] with a CHK2-inhibitor complex (PDB code 2WTJ) with the inhibitor and water molecules removed as a search model. The protein-ligand structures were manually rebuilt in COOT [78] and refined with BUSTER [79] in iterative cycles. Ligand restraints were generated with Grade [80] and Mogul [81]. The positioning of the furan ring in compound **13** was guided using Isostar maps [82] calculated using data from the Cambridge Structural database and from the protein databank respectively. The quality of the structures was assessed with MOLPROBITY [83]. The coordinates of compounds **11**–**19** and compounds **20** and **22** and their associated structure factors have been deposited in the Protein Data Bank with accession codes, 4BDA, 4BDB, 4BDC, 4BDD, 4BDE, 4BDF, 4BDG, 4BDH, 4BDI and 4BDJ, 4BDK, respectively.

## Supporting Information

Figure S1Assay performance in the fragment screen. Assay reproducibility for the screen in triplicate for all fragments. The mean values for the total activity (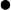), no enzyme blanks (◼) and robust Z’ (▲) are shown. There were 320 compounds on each plate.(TIF)

Figure S2 IC_50_ values of the mutual AlphaScreen™ and thermal shift hits compared with the three most prominent Tm-shift hits classed as inactives in the AlphaScreen™. The figure shows that the three latter compounds (category 3, shown in orange) have IC_50_ values significantly higher than the mutual hits (category 1, shown in red), consistent with the primary screening data and are therefore less attractive to follow up. Square symbols denote compounds for which co-crystal structures with trCHK2 were determined (see Table S1). The IC_50_ values are indicated as mean ± standard deviation from triplicate measurements.
(TIF)

Figure S3 Chemical structures of the spleen tyrosine kinase inhibitor (**24**,) the JNK3 inhibitor SR3451 (**25**) and the early arylbenzimidazole inhibitor compound (**26)**.
(TIF)

Figure S4 Chemical structures of positive controls (compounds **27** and **28**) used in AlphaScreen™ and mobility shift assays.
(TIF)

Table S1 Crystallographic data collection and refinement statistics for fragment hits and follow-up compounds.
(DOC)
