# Supplementary figures and images for: Fragment-Based Screening Maps Inhibitor Interactions in the ATP-Binding Site of Checkpoint Kinase 2
Source: PLoS One. 2013 Jun 12;8(6):e65689. doi: 10.1371/journal.pone.0065689 (PMC3680490; doi:10.1371/journal.pone.0065689)

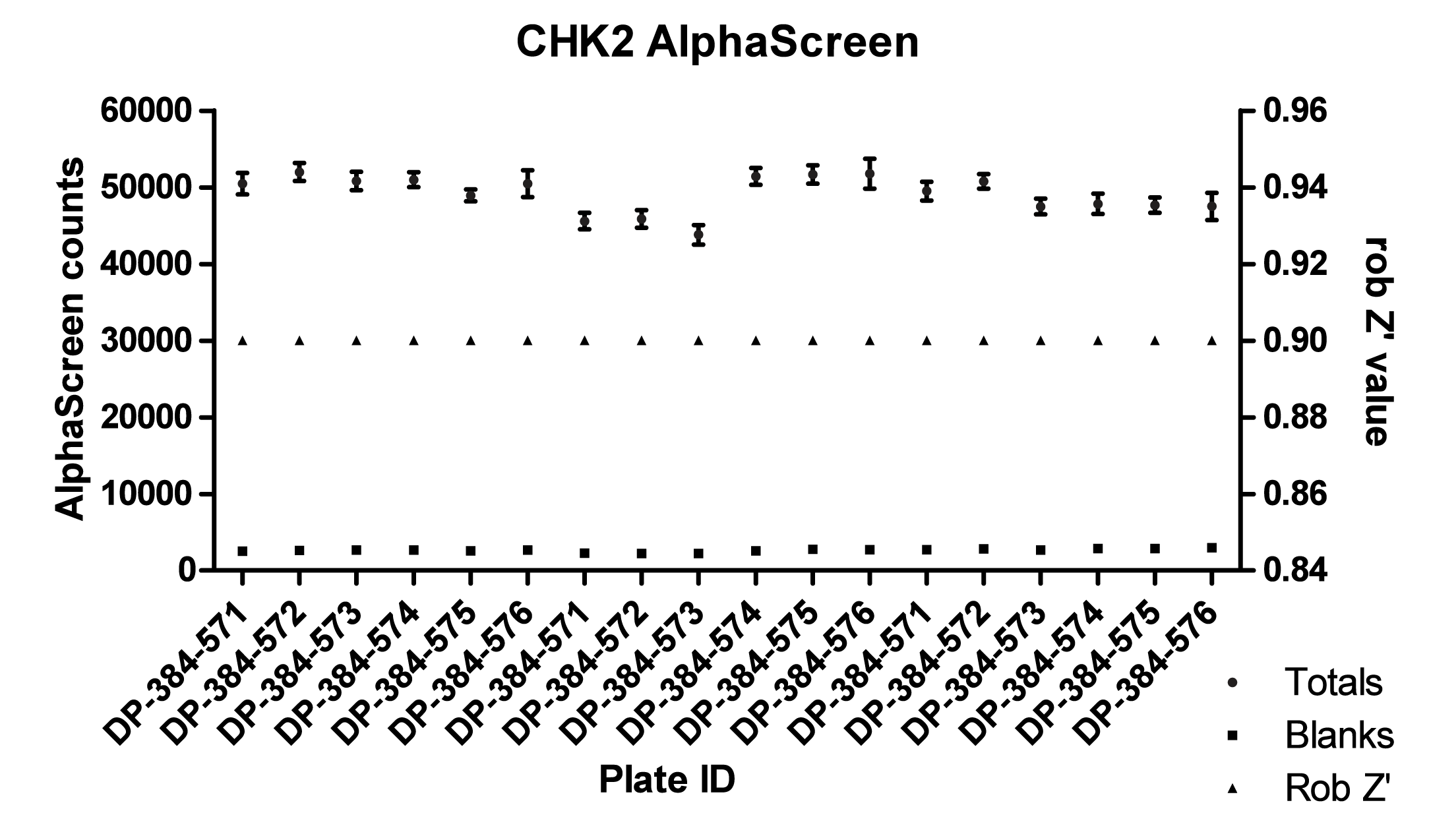

Supplement: Figure S1 — Assay performance in the fragment screen. Assay reproducibility for the screen in triplicate for all fragments. The mean values for the total activity (), no enzyme blanks (◼) and robust Z’ (▲) are shown. There were 320 compounds on each plate. (TIF) [file pone.0065689.s001.tif]

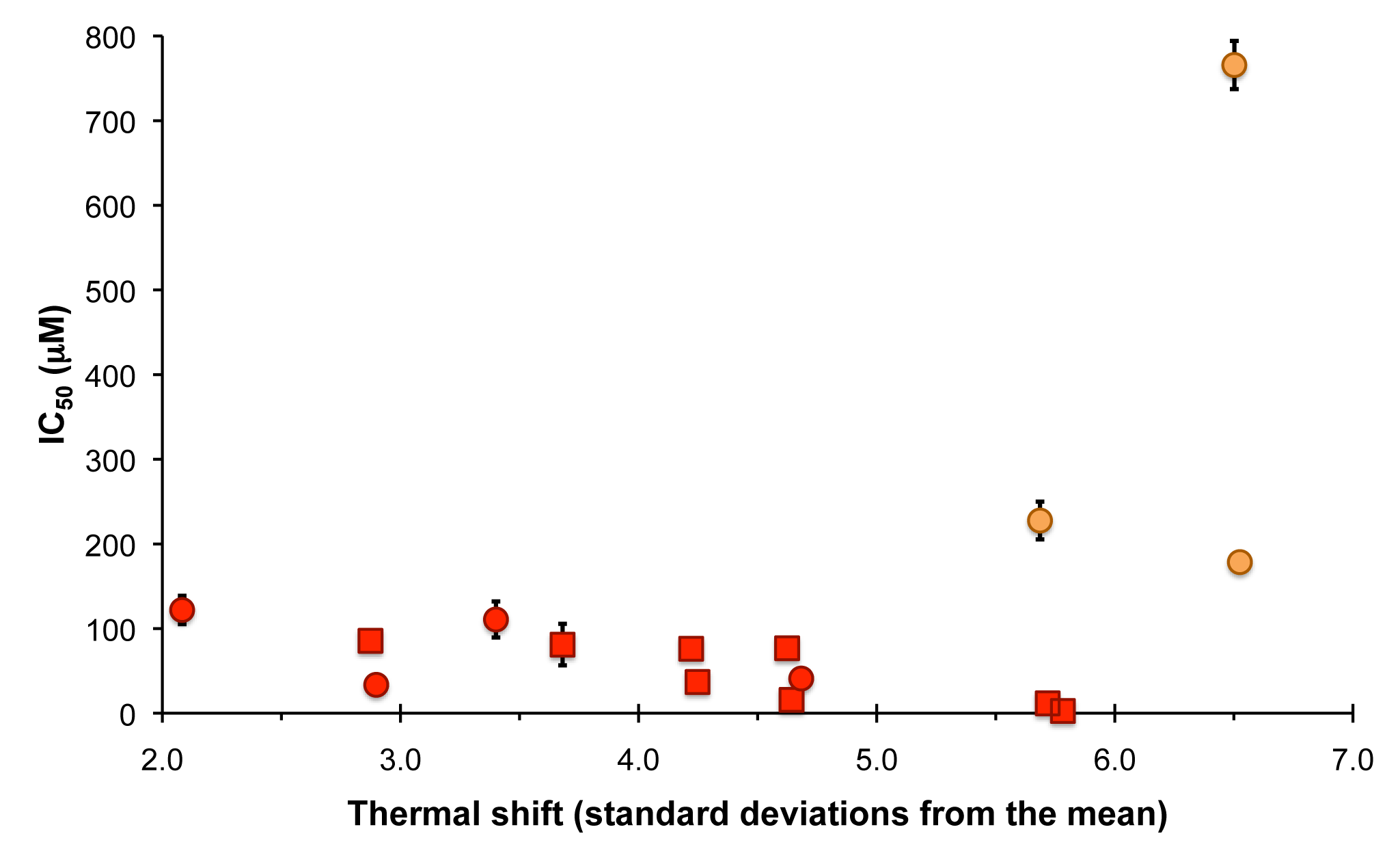

Supplement: Figure S2 — IC50 values of the mutual AlphaScreen™ and thermal shift hits compared with the three most prominent Tm-shift hits classed as inactives in the AlphaScreen™. The figure shows that the three latter compounds (category 3, shown in orange) have IC50 values significantly higher than the mutual hits (category 1, shown in red), consistent with the primary screening data and are therefore less attractive to follow up. Square symbols denote compounds for which co-crystal structures with trCHK2 were determined (see Table S1). The IC50 values are indicated as mean ± standard deviation from triplicate measurements. (TIF) [file pone.0065689.s002.tif]

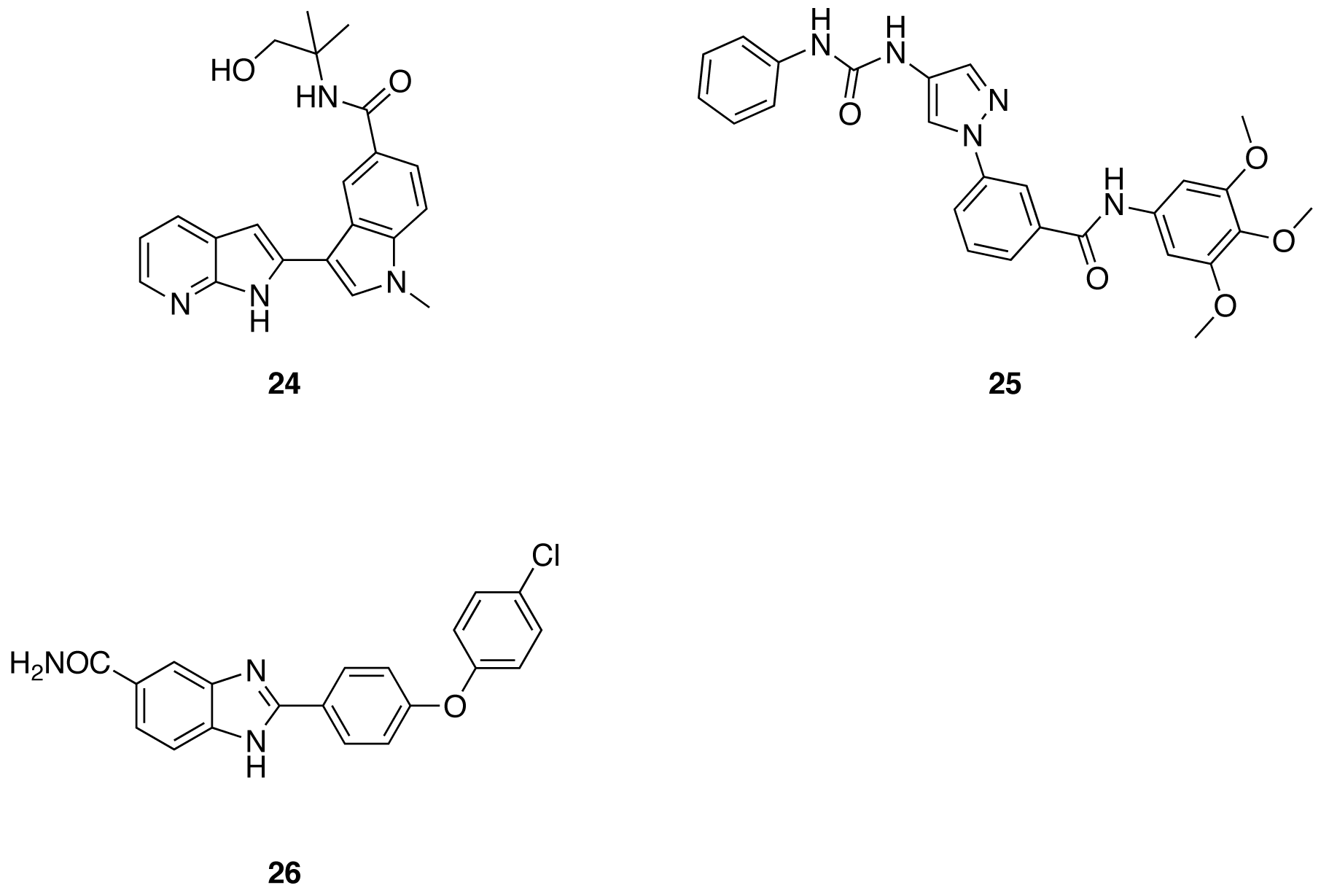

Supplement: Figure S3 — Chemical structures of the spleen tyrosine kinase inhibitor (24,) the JNK3 inhibitor SR3451 (25) and the early arylbenzimidazole inhibitor compound (26). (TIF) [file pone.0065689.s003.tif]

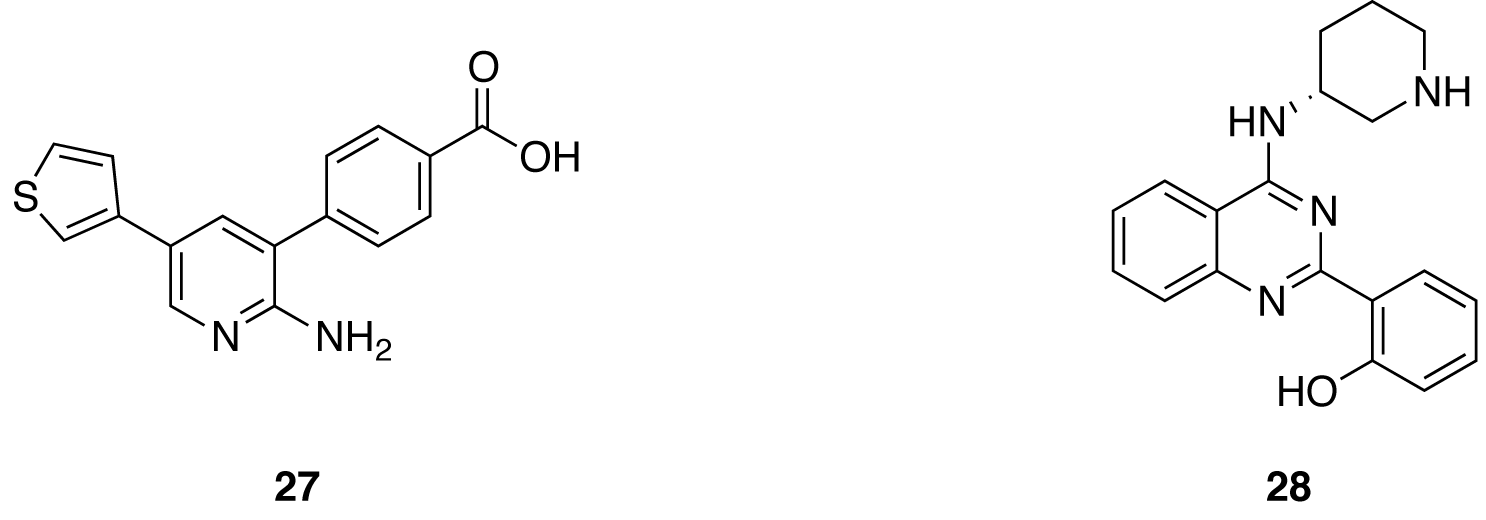

Supplement: Figure S4 — Chemical structures of positive controls (compounds 27 and 28) used in AlphaScreen™ and mobility shift assays. (TIF) [file pone.0065689.s004.tif]
