# Supplementary material for: Fragment-Based Screening Maps Inhibitor Interactions in the ATP-Binding Site of Checkpoint Kinase 2
Source: PLoS One. 2013 Jun 12;8(6):e65689. doi: 10.1371/journal.pone.0065689 (PMC3680490; doi:10.1371/journal.pone.0065689)
Supplement: Table S1 — Crystallographic data collection and refinement statistics for fragment hits and follow-up compounds. (DOC) [file pone.0065689.s005.doc]

**Table S1.** Data collection and refinement statistics.

| *Ligand* | Compound **11** | Compound **12** | Compound **13** | Compound **14** |
| --- | --- | --- | --- | --- |
| *Crystals* |  |  |  |  |
| Space group | P3_2_21 | P3_2_21 | P3_2_21 | P3_2_21 |
| Lattice constants |  |  |  |  |
| a (Å) | 91.13 | 91.13 | 91.1 | 91.16 |
| b (Å) | 91.13 | 91.13 | 91.1 | 91.16 |
| c (Å) | 93.11 | 92.91 | 93.19 | 92.77 |
| α (°) | 90 | 90 | 90 | 90 |
| β (°) | 90 | 90 | 90 | 90 |
| γ (°) | 120 | 120 | 120 | 120 |
| *Data collection* |  |  |  |  |
| Diamond Beamline | I04 | I02 | I04 | I02 |
| Wavelength (*λ*) | 0.97280 | 0.97960 | 0.97280 | 0.97960 |
| Resolution range (Å) | 40.10-2.60 | 60.15-2.5 | 40.92-3.00 | 60.08-2.67 |
| (highest-resolution shell values) | (2.74-2.60) | (2.64-2.5) | (3.16-3.00) | (2.81-2.67) |
| Unique reflections | 14123 (2023) | 15700 (2246) | 9297 (1345) | 12966 (1883) |
| Completeness (%) | 99.8 (99.9) | 99.3 (99.7) | 99.9 (100.0) | 99.3 (100.0) |
| Multiplicity | 3.5 (3.6) | 5.4 (5.5) | 6.0 (6.2) | 5.3 (5.4) |
| R_merge_ (%) | 7.9 (48.3) | 6.4 (49.5) | 9.1 (48.9) | 6.8 (44.5) |
| I/σ(I) | 7.2 (1.6) | 7.7 (1.5) | 6.3 (1.6) | 7.0 (1.6) |
| Mean I/σ(I) | 10.6 (2.6) | 13.9 (2.0) | 11.2 (3.3) | 15.4 (3.5) |
| *Refinement* |  |  |  |  |
| No. of amino acids | 284 | 283 | 283 | 281 |
| No. of water molecules | 91 | 93 | 15 | 46 |
| No. of NO_3_ ions | 1 | 1 | 1 | 1 |
| No. of Cl^-^ ions | 1 | 0 | 1 | 0 |
| No. of ethylene glycol molecules | 7 | 9 | 1 | 3 |
| R_work_ (%) | 18.0 | 19.1 | 16.8 | 18.7 |
| R_free_ (%) | 23.7 | 22.4 | 21.3 | 22.0 |
| *Ramachandran plot* |  |  |  |  |
| Favoured (%) | 97.1 | 97.1 | 96.0 | 95.6 |
| Outliers (%) | 0.0 | 0.0 | 0.0 | 0.0 |
| RMSD bonds (Å) | 0.010 | 0.009 | 0.010 | 0.009 |
| RMSD angles (°) | 1.140 | 1.100 | 1.140 | 1.100 |
| PDB ID | 4BDA | 4BDB | 4BDC | 4BDD |

**Table S1 Continued.**

| *Ligand* | Compound **15** | Compound **16** | Compound **17** | Compound **18** |
| --- | --- | --- | --- | --- |
| *Crystals* |  |  |  |  |
| Space group | P3_2_21 | P3_2_21 | P3_2_21 | P3_2_21 |
| Lattice constants |  |  |  |  |
| a (Å) | 91.08 | 90.95 | 90.57 | 91.08 |
| b (Å) | 91.08 | 90.95 | 90.57 | 91.08 |
| c (Å) | 93.32 | 93.03 | 92.67 | 92.96 |
| α (°) | 90 | 90 | 90 | 90 |
| β (°) | 90 | 90 | 90 | 90 |
| γ (°) | 120 | 120 | 120 | 120 |
| *Data collection* |  |  |  |  |
| Diamond Beamline | I02 | I04 | I04 | I02 |
| Wavelength (*λ*) | 0.97960 | 0.97280 | 0.97280 | 0.97960 |
| Resolution range (Å) | 60.19-2.55 | 78.81-2.70 | 78.33-2.84 | 60.14-2.70 |
| (highest-resolution shell values) | (2.69-2.55) | (2.85-2.70) | (2.99-2.84) | (2.85-2.70) |
| Unique reflections | 14972 (2142) | 12578 (1814) | 10741 (1542) | 12574 (1818) |
| Completeness (%) | 99.8 (100.0) | 99.8 (99.9) | 99.9 (100.0) | 99.5 (100.0) |
| Multiplicity | 5.9 (6.1) | 3.5 (3.6) | 5.1 (5.3) | 4.7 (4.8) |
| R_merge_ (%) | 7.9 (45.8) | 9.9 (47.3) | 8.0 (50.3) | 6.7 (41.0) |
| I/σ(I) | 6.9 (1.5) | 4.6 (1.6) | 6.6 (1.5) | 7.5 (1.8) |
| Mean I/σ(I) | 14.8 (4.4) | 8.7 (3.0) | 11.2 (3.0) | 15.5 (3.6) |
| *Refinement* |  |  |  |  |
| No. of amino acids | 283 | 285 | 286 | 282 |
| No. of water molecules | 82 | 50 | 34 | 65 |
| No. of NO_3_ ions | 1 | 1 | 1 | 1 |
| No. of Cl^-^ ions | 0 | 0 | 1 | 1 |
| No. of ethylene glycol molecules | 4 | 2 | 1 | 3 |
| R_work_(%) | 18.5 | 18.1 | 18.6 | 17.7 |
| R_free_ (%) | 21.5 | 23.3 | 22.7 | 22.5 |
| *Ramachandran plot* |  |  |  |  |
| Favoured (%) | 96.7 | 96.4 | 96.4 | 96.7 |
| Outliers (%) | 0.0 | 0.0 | 0.0 | 0.0 |
| RMSD bonds (Å) | 0.009 | 0.010 | 0.010 | 0.010 |
| RMSD angles (°) | 1.050 | 1.160 | 1.140 | 1.140 |
| PDB ID | 4BDE | 4BDF | 4BDG | 4BDH |

**Table S1 Continued.**

| *Ligand* | Compound **19** | Compound **20** | Compound **22** |
| --- | --- | --- | --- |
| *Crystals* |  |  |  |
| Space group | P3_2_21 | P3_2_21 | P3_2_21 |
| Lattice constants |  |  |  |
| a (Å) | 90.23 | 90.93 | 91.01 |
| b (Å) | 90.23 | 90.93 | 91.01 |
| c (Å) | 92.9 | 92.13 | 92.84 |
| α (°) | 90 | 90 | 90 |
| β (°) | 90 | 90 | 90 |
| γ (°) | 120 | 120 | 120 |
| *Data collection* |  |  |  |
| Diamond Beamline | I24 | I24 | Bruker Microstar |
| Wavelength (*λ*) | 0.96861 | 0.96861 | 1.54189 |
| Resolution range (Å) | 39.07-2.31 | 36.21-3.01 | 45.5-3.30 |
| (highest-resolution shell values) | (2.37-2.31) | (3.09-3.01) | (3.40-3.30) |
| Unique reflections | 19611 (1412) | 9073 (665) | 6975 (579) |
| Completeness (%) | 99.9 (100.0) | 99.9 (99.9) | 99.5 (100.0) |
| Multiplicity | 9.2 (9.7) | 8.5 (8.4) | 8.3 (7.1) |
| R_merge_ (%) | 4.1 (57.9) | 5.7 (67.2) | 10.8 (29.3) |
| I/σ(I) | 9.0 (1.3) | 8.3 (0.9) | - |
| Mean I/σ(I) | 24.4 (3.9) | 22.9 (3.4) | 8.7 (3.4) |
| *Refinement* |  |  |  |
| No. of amino acids | 290 | 282 | 286 |
| No. of water molecules | 53 | 4 | 28 |
| No. of NO_3_ ions | 1 | 1 | 1 |
| No. of Cl^-^ ions | 1 | 0 | 0 |
| No. of ethylene glycol molecules | 7 | 0 | 3 |
| R_work_ (%) | 18.8 | 17.7 | 16.8 |
| R_free_ (%) | 22.1 | 21.5 | 23.3 |
| *Ramachandran plot* |  |  |  |
| Favoured (%) | 96.5 | 96.7 | 96.4 |
| Outliers (%) | 0.0 | 0.0 | 0.4 |
| RMSD bonds (Å) | 0.010 | 0.010 | 0.010 |
| RMSD angles (°) | 1.090 | 1.140 | 1.190 |
| PDB ID | 4BDI | 4BDJ | 4BDK |
